# Racial/Ethnic and Gender Inequities in the Sufficiency of Paid Leave During the COVID-19 Pandemic: Evidence from the Service Sector Inequities in Paid Leave Sufficiency

**DOI:** 10.1002/ajim.23533

**Published:** 2023-08-28

**Authors:** Julia M. Goodman, Daniel Schneider

**Affiliations:** a Oregon Health & Science University—Portland State University School of Public Health; b Harvard University, Harvard Kennedy School

**Keywords:** paid leave, social determinants of health, service-sector workers, COVID-19

## Abstract

**Background:**

Access to paid family and medical leave (PFML), including leave to care for a seriously-ill loved one or recover from one’s own serious illness, conveys health and economic benefits for workers and their families. However, without a national PFML policy, access to paid leave remains limited and unequal. Previous work documenting inequitable access by socioeconomic status and race/ethnicity primarily focuses on parental leave, measures theoretical access to paid leave rather than actual leave uptake, and lacks an accounting for why workers of color and women may have less access to PFML. We extend this literature by looking at leave-taking for medical needs or caregiving among a high-risk population during the COVID-19 pandemic.

**Methods:**

We draw on data from 2,595 service-sector workers surveyed by the Shift Project in 2020 and 2021 to estimate inequities in leave uptake among workers who experienced qualifying events. We then estimate the relative importance of worker demographic characteristics, qualifying event types (medical vs. caregiving leave), proxies for access to state and employer PFML policies, job characteristics, and ultimately within-firm differences to these gaps.

**Results:**

Overall, one-fifth of workers reported sufficient leave. Women are significantly more likely than men to report insufficient or no leave. Hispanic and Black workers are more likely to take insufficient or no leave, respectively, but these differences were attenuated when controlling for covariates.

**Conclusions:**

The dearth of PFML laws leaves women and workers of color without access to leave that is paid and of sufficient duration when facing a qualifying event.

## 1. INTRODUCTION

The COVID-19 global pandemic has laid bare the critical public health importance of access to paid leave, resulting in expanded paid leave policy efforts at the local, state, and federal levels. In a context where 41% of US households report not having enough savings to cover a $2,000 financial shock,^1^ workers who lacked paid leave, but attempted to follow public health guidance to stay home when sick, faced significant financial penalties including potential job loss. The acute challenges of managing COVID-19 exposure and illness added to those long experienced by workers who may need extended periods of paid time off to recover from serious illness or injury. Similarly, the COVID-19 pandemic has further underscored the particular challenges faced by workers with caregiving responsibilities who require leave not only for their own illness, but also to care for sick family members.

Paid family and medical leave (PFML) laws are designed to allow workers to take extended periods of leave for caregiving or serious health conditions. Most state programs are funded through payroll contributions (employers and/or employees) and administered by the state.^2^ Eligibility is based on minimum contributions or hours worked in a base period; some states require a waiting period before benefits are paid. PFML laws are complementary to but distinct from paid sick leave laws, which are designed for acute needs, although most state PFML laws allow leave to be taken in increments of one day. The maximum leave available ranges from six weeks for family caregiving leave (Rhode Island, District of Columbia) to 52 weeks for one’s own disability (California).

The weight of prior research on PFML has focused on parental leave and there is a vast body of evidence linking paid leave for new parents with improved health and well-being.^3–10^ However, PFML also importantly covers leave to care for a seriously ill loved one or to recover from one’s own serious illness, and such reasons for leave are far more common than parental leave.^11^ Effective access to medical and caregiving paid leave has been tied to a range of health and economic benefits for workers and their families even outside the pandemic. Among workers facing medical or caregiving needs, those who take paid leave report improved financial security and well-being relative to those who do not take paid leave.^12^ Paid leave is associated with reduced financial hardship and improvements in self-reported quality of life for patients experiencing a serious medical condition (i.e., bone marrow transplantation).^13^ Paid family leave may reduce nursing home utilization among older adults, presumably by freeing up time for family caregivers.^14^

During the peak of the COVID-19 pandemic, the Families First Coronavirus Response Act (FFCRA) and subsequent extension through the American Rescue Plan Act (ARPA) required covered employers to provide two weeks of fully-paid sick leave to employees unable to work because they are quarantined and/or experiencing COVID-19 symptoms and two weeks of partially-paid sick leave to employees unable to work because they need to care for someone in quarantine or a child whose school or childcare center is closed due to COVID-19.^15^ Employees who had been employed for at least 30 days were entitled to an additional 10 weeks of paid expanded family and medical leave at two-thirds the employee’s regular rate of pay to care for a child whose school or childcare provider is closed due to COVID-19. However, the FFCRA exempted firms with fewer than 50 or more than 499 employees. Since 1993, the Family and Medical Leave Act (FMLA) has provided job-protected, unpaid caregiving leave to covered workers, but strict eligibility requirements exclude about half of the workforce.^11^ A growing number of states (CA, NJ, RI, NY, WA, MA, CT, OR, CO, MD, DE, MN, ME and DC) now have paid family and medical leave laws;^16^ still, the majority of workers rely on their employers to voluntarily provide these benefits.

Together, these policies and practices set up a system where individual, employer, and state policy characteristics may each inform the likelihood a given worker can use PFML. This incomplete patchwork raises the risk that access to medical and to caregiving paid leave may not only be limited, but may also be unequal along the lines of race/ethnicity and gender. Women and workers of color face heightened caregiving responsibilities and unique health challenges, yet are also disadvantaged at work, which may constrain effective access to needed PFML.^17–20^

Where prior research focuses on racial/ethnic inequalities in potential access to parental leave, we advance this work by focusing specifically on caregiving and medical leave, examining the COVID-19 period, deploying a novel measure of the sufficiency of leave actually taken (rather than hypothetical access to leave or the use of any leave), documenting racial/ethnic and gender disparities in access, and decomposing these gaps. To do so, we leverage a large, national sample of service and retail workers surveyed during the COVID-19 pandemic that contains uniquely rich data on leave taking in an employer-employee matched sample.

### 1.1 Inequities in Access to Paid Leave

According to the U.S. Bureau of Labor Statistics’ 2022 National Compensation Survey, 25% of civilian workers (public and private sector) have access to paid family leave through their jobs, but this number obscures huge disparities by worker and employer characteristics.^21^ For example, full-time workers are more than twice as likely to have paid family leave as part-time workers, and workers in the top decile of earnings are almost six times more likely to have paid family leave than those in the lowest earnings decile. Nationally, one-third of workers in management, professional, and related occupations have access to paid family leave, compared to just 16% of those in service occupations.^22^ In a study of new parents in the San Francisco Bay Area, workers in leisure and hospitality were 17% less likely to receive full pay during leave than those in professional and financial services, and received 1.6 fewer full-pay equivalent weeks of leave.^23^ At the same time, service-sector workers found themselves on the front lines of the COVID-19 pandemic response and bore a significant risk of exposure to infection.

Emerging evidence also suggests that access to PFML varies substantially by race/ethnicity. Using data from the 2011 American Time Use Survey (ATUS) Leave Module, Bartel et al. (2019) report that Black and Hispanic workers are significantly less likely than non-Hispanic white workers to report that they could access paid parental and family caregiving leave in unadjusted models, and significant differences between white, non-Hispanic and Hispanic workers remain after controlling for occupational and sociodemographic characteristics.^24^ Drawing on the more recent 2017–18 ATUS Leave Module, Goodman et al. (2022) find that both Black and Latinx workers are less likely than white workers to report access to paid parental, family caregiving, and medical leave, even after controlling for occupational and sociodemographic characteristics.^25^ Even when paid leave is offered, it may not be equivalent across racial and ethnic groups: one study of new parents in the San Francisco Bay Area found that Black, Latinx, and Asian workers have fewer fully-paid weeks of leave after the birth of a child than their white counterparts.^26^

Reported access to paid leave does not necessarily result in uptake when needed. Evidence for racial and ethnic inequities in actual use of paid leave is mixed, and may depend on the reason for needing leave. Bartel et al. (2019) show small and mostly non-significant racial/ethnic differences in use of paid maternity leave using data from the Survey of Income and Program Participation (SIPP) and in use of paid maternity or paternity leave using data from the Current Population Survey (CPS).^24^ Goodman et al. (2021) find no difference in the amount of leave taken after childbirth by new parents across racial and ethnic groups, despite significant differences in the amount of pay received.^26^ However, neither of these studies examines leave-taking for caregiving or medical reasons, which may differ from parental leave. Brown et al. (2020), using the 2018 FMLA Surveys to examine unpaid, job-protected leave-taking, suggest that limited access to paid leave may contribute to a higher unmet need for leave (meaning a worker experienced a life event that would qualify them for job-protected leave under the FMLA but did not take leave): 11% of Black private-sector workers (compared to 6% of white workers) had an unmet need for leave in the past 12 months.^27^

Even less is known about difference in paid leave access and uptake by gender, particularly for reasons other than parental leave. Limited evidence suggests that female workers are more likely to report an unmet need for leave than male workers.^27^ Women are also significantly more likely to report a lack of access to paid family and medical leave of any kind than men, but are especially less likely to have access to eldercare or childcare leave.^24^

In all, the literature suggests more limited access to paid leave for Black and Hispanic workers across types of qualifying events, with mixed evidence about actual leave-taking and limited evidence about gender inequities in access or uptake.

### 1.2 Sources of Inequity

While inequitable access to paid leave is apparent, the reasons for it are less clear and, again, the literature focuses more on access than uptake. One possibility for inequitable access is that occupational segregation sorts women and workers of color into jobs that are less likely to provide benefits, including paid leave. Relative to white and Asian workers, Black and Latinx workers are underrepresented in professional-class jobs, which are more likely to provide benefits like paid leave.^28,29^ Controlling for other characteristics, women, Black, Hispanic, and foreign-born working parents are significantly more likely to hold jobs that provide no health insurance, no pension, and that pay below a family economic security wage.^29^ While a compensating differentials perspective would suggest that women and workers of color may receive lower wages and fewer economic benefits in exchange for other job amenities, such as paid leave,^30,31^ current research instead finds little evidence of such compensating differentials^32,33^ and instead it appears that women and workers of color are segregated into jobs that are “low road” on multiple dimensions of quality.^34^

Another possibility is that women and workers of color may be less likely to hold jobs that qualify them for paid leave benefits, either by working part-time or having short job tenure (both employer-provided and state paid leave policies often carry minimum hours or job tenure requirements). Evidence here is mixed, and may vary by stage of the life course. For example, Hispanic workers overall are equally likely to work full-time as non-Hispanic white workers, but this is not necessarily the case among working parents.^28^ In contrast, there is some evidence that gender inequalities in access to any paid family or medical leave are due in part to gender differences in part time status, union membership, and occupation.^24^

Additionally, given the patchwork of state and local PFML laws in the United States, it is also possible that the geographic segregation of workers of color could drive inequalities in leave-taking in response to a qualifying event. In particular, the concentration of Black workers in the South, where such PFML laws have not been enacted, could contribute to inequalities in leave taking. However, these dynamics of segregation should not contribute to gender inequalities in leave-taking.

In the most comprehensive assessment of the relative importance of these mechanisms to date, Bartel et al. (2019) conducted a Oaxaca decomposition to understand what was driving observed differentials in paid leave access among Hispanics and non-Hispanic whites and found that both average sociodemographic and occupational characteristics and returns to those characteristics played a role. In particular, they point to differences in immigration or citizen status and lower returns to working full time as contributing to these differentials.^24^ While a significant contribution to our understanding of the sources of inequalities in leave taking, this work focuses on the qualifying event of a new child, and does not examine inequalities in leave-taking for caregiving and medical reasons nor does it decompose gender gaps in access to leave-taking of any kind.

Even where paid leave policies exist, however, discrimination may contribute to less uptake of paid leave benefits among people of color and among women. The downstream effects of racism and sexism could include increased stigma around leave-taking, fear of retribution, or lack of awareness of paid leave benefits. One study of new parents in the San Francisco Bay Area found that Black and Latinx new parents were significantly less likely to understand their maternity leave benefits than white workers, and their employers were perceived as less helpful in making sure they understood them.^26^ Female workers might seek to avoid stigma around leave taking that is produced by the conflict between the schemas of “family devotion” and the role of primary caretaker with that of the ideal, devoted worker.^35–38^ Despite bearing a disproportionate share of care work,^39,40^ female workers might, in some ways as a result, actually be less likely to take up needed leave, particularly for medical or family caregiving that could be perceived as less of a necessity than leave for childbirth. This form of on-the-job discrimination^41^ is difficult to measure directly, but would be consistent with residual race/ethnicity and/or gender gaps in leave-taking after adjusting for observable characteristics.

Previous studies have examined racial/ethnic inequities in paid leave access^25^ and in use of parental leave after childbirth.^23^ The current study extends the literature by focusing on gender and racial/ethnic inequities in the self-reported sufficiency of leave taken among workers who needed it for family caregiving or medical needs. In contrast to reported access to paid leave, which may suffer from response bias among respondents who have not needed to take leave and therefore may not be aware of available leave, leave-taking among people who experienced a qualifying event helps us understand inequities in who is able to actually take leave when needed. Further, this study narrows in on service and retail workers – a group that represents over 17% of the U.S. labor force and that often lacks the capacity to take paid family or medical leave when needed.^12^ Finally, where prior work as relied on data from 2018 and earlier, we draw on data collected during the COVID-19 pandemic, when the need for paid medical and caregiving leave, especially among frontline workers, was especially pronounced.

## 2. DATA & METHODS

### 2.1 Data

We draw on new data collected by the Shift Project between September of 2020 and November of 2021 from 8,212 hourly service-sector workers employed at 111 large firms who reported experiencing a PFML-qualifying event in the 12 months preceding interview. A full list of these firms is provided in Appendix Table A-I.

Our data are drawn using a novel sampling approach. The Shift Project uses Facebook/ Instagram as both quasi-sampling framing and recruitment device, using Facebook’s sophisticated ad targeting system to construct “audiences” of workers at specific large named service-sector firms. The Shift Project then recruits these workers to the online survey by fielding paid advertisements that appear in workers’ newsfeeds on desktop and mobile. This approach is low-cost, very flexible, and allows for rapid-response data collection. In this instance, The Shift Project approach allowed for the mid-pandemic collection of detailed data on qualifying events and leave taking alongside economic outcomes for a large sample of vulnerable workers. However, these data are drawn using a non-probability sampling approach. Prior methodological work finds that univariate distributions and multi-variate associations in Shift replicate those in “gold-standard” data sources such as the NLSY and Current Population Survey^42^ and these data have been used to examine the correlates and consequences of precarious job quality.^34,43,44^ In these analyses, we weight the Shift Project sample to the characteristics of workers in the same occupations and industries in the American Community Survey on race/ethnicity, gender, and age and employ these weights in all of our estimates.

The Shift Project collects biannual repeated cross sections of survey data from hourly workers employed at large firms in the US service sector. These data have been collected over 11 waves, through the Fall of 2021. We draw on data collected at Waves 9 through 11, which included detailed questions about paid family and medical leave.

All survey respondents are asked if they experienced any of three types of events that would “qualify” them for paid leave under most existing laws and company policies. Respondents were asked if they (1) “welcomed a new child into their family through birth, adoption, or foster placement,” (2) “had a serious health condition or illness, like recovering from a surgery or serious illness,” and (3) “have needed to care for seriously ill or injured family member.” For each item, respondents were asked about their experience with each event over the prior 12 months. Respondents could report more than one type of event. In this analysis, we focus on medical and caregiving events, dropping 599 respondents who reported a new child as their only qualifying event. We exclude respondents who experienced a qualifying event but reported that they did not take leave because they did not need to take time off.

Our analytic sample includes 2,595 respondents who had a serious health condition or illness, like recovering from a surgery or serious illness, or needed to care for seriously ill or injured family member in the recall period and had complete data. For each item, respondents interviewed between September and November of 2020 (Wave 9) were asked about their experience with each event since January 1 of 2020; respondents interviewed between March and May of 2021 (Wave 10) and September and November of 2021 (Wave 11) were asked about the prior 12 months.

### 2.2 Measures

Among respondents who experienced a qualifying event and wanted to take leave, we construct a variable to measure whether respondents were satisfied with their available leave (Leave Sufficiency). We separately examine whether they received pay (Leave Compensation), reported in the Appendix. While one might expect that sufficient leave is equivalent to paid leave (and insufficient leave equivalent to unpaid leave), this is not true in our data. The leave sufficiency and leave compensation measures overlap (by design, both include a category of those who took no leave), but are distinct. For example, 60% of workers reporting sufficient leave had no paid leave. On the other hand, 29% of workers reporting insufficient leave did have paid leave (Appendix Table A-II).

#### Leave Sufficiency.

Respondents who reported at least one qualifying event were asked if they took leave from their job in response. Respondents who did not take any leave were asked why they did not take leave. Those who reported that they did not need any leave were dropped from the sample. Those who reported a barrier to leave-taking (could not afford any leave, pressure from employer, afraid of losing job, concerned about losing health insurance, not knowing leave was an option) were coded as not taking leave, but wanting leave. Respondents who took leave were asked why they did not take more leave. Those who reported that they returned to work because they no longer needed to be away from work were characterized as taking leave of sufficient length. Those who reported that they returned to work because of a barrier to additional leave-taking (same list of reasons as above) were characterized as taking leave of insufficient length. Together, these questions uniquely allow us to categorize respondents who experienced a qualifying event that necessitated some leave taking into three mutually exclusive categories: (1) did not take leave, but wanted leave; (2) took leave, but wanted more (insufficient length); and (3) took leave, and did not want more (sufficient length).

#### Leave Compensation.

Respondents who took leave were asked if they received pay from their employer, with options of receiving full pay, partial pay, or no pay. We combine this measure with whether they took any leave to code respondents into three mutually exclusive categories: (1) did not take any leave; (2) took unpaid leave only; (3) took paid leave. We report results of models using this outcome variable in the Appendix.

Our key predictors are respondent gender (men; women) and race/ethnicity (white, non-Hispanic; Black, non-Hispanic, Hispanic; other or multiple race/ethnicities, non-Hispanic).

We measure and control for a set of demographic characteristics: marital status (single, cohabiting, married); age; having children ages 0 to 4, ages 5 to 9, ages 10 to 14, and ages 15 to 18; current school enrollment; and educational attainment (< HS; HS/GED; some college; Associates degree; Bachelors degree; Masters degree or more). We control for the type of qualifying event (own health; caregiving; multiple events). We also measure and control for a set of additional characteristics that could affect access or eligibility for paid leave: job tenure, union coverage, hourly wage, number of usual work hours, and state. We also measure and control for occupation and employer to account for segregation. Finally, we include a set of month and year fixed-effects.

### 2.3 Models

We estimate multinomial logistic regression models for each of our main outcomes. Because our prior assumption is that there will be differences in both leave sufficiency and leave compensation, we build a series of stepwise regression models to better understand what is driving any such gaps. We first present (1) unadjusted models and then models that adjust for (2) demographics (marital status, age, age of children, current school enrollment, and educational attainment), (3) type of qualifying event, (4) access or eligibility for leave (job tenure, union coverage, hourly wage, number of usual work hours, and state), (5) and occupation and employer. We include month and year fixed-effects in all models. All analyses are conducted in Stata 14.2 ^45^.

We then use Blinder-Oaxaca decompositions using the Oaxaca command in order to understand whether differences in leave sufficiency result from differences in “endowments” (i.e., average characteristics), returns on those endowments (i.e., regression coefficients), or the interaction between endowments and coefficients ^46,47^. Because the outcome and comparison groups both need to be binary, we run a series of decompositions by gender (male vs. female) and race (Black vs. white), separately for insufficient vs. sufficient leave and no leave vs. sufficient leave.

## 3. RESULTS

A minority of respondents who experienced qualifying events that would have merited leave-taking were satisfied with their leave situation: 21.2% took sufficient leave, meaning they took leave and did not want to take more (Table I). 38.4% of respondents took insufficient leave (took leave but wanted more) and 40.4% did not take any leave, but wanted to. 40.2% of respondents took unpaid leave only; 19.4% took paid leave.

Table II shows occupational and demographic characteristics of the sample. Three-quarters of the sample identified as women. Eighty-five percent were non-Hispanic white, 3% non-Hispanic Black, 7% Hispanic of any race, and 5% as other or multiple races.

In the unadjusted model and when controlling for demographic characteristics and reason for taking leave, women were significantly more likely than men to take insufficient leave and to take no leave, relative to taking sufficient leave (Table III). As additional controls for access were added to the models, the increased likelihood of taking insufficient leave was attenuated, but remained statistically significant, whereas women were no longer more likely to take no leave. Once occupation and employer fixed effects were added, women were no longer significantly more likely to take insufficient leave.

In the unadjusted model, Hispanic workers were significantly more likely to take insufficient leave compared to sufficient leave and non-Hispanic Black workers were significantly more likely than white workers to take no leave when needed, but these relationships attenuated in multivariate models (Table III).

Similar patterns emerge when examining leave compensation (Appendix Table A-III). In unadjusted models and models that control for demographics and event type, women were significantly more likely than men to take unpaid leave and no leave when needed, relative to taking paid leave. Once access, occupation and employer were included in the models, the coefficients were reduced. Women were no longer more likely to take no leave when needed or to take unpaid leave, as compared to taking paid leave. In contrast, adding controls strengthens the relationship with taking unpaid leave and, to a lesser extent, no leave among non-Hispanic Black workers. In fully adjusted models, non-Hispanic Black workers were more likely to take unpaid and no leave, as compared to white workers.

Blinder-Oaxaca decompositions help us further interrogate these relationships. Tables IV-V summarize the results of these decompositions by gender (Table IV) and race (non-Hispanic Black vs. white, Table V). Women’s increased likelihood of taking no leave when needed appears to be driven by differences in endowments; in particular, differences in demographics and occupational characteristics. The increased likelihood of taking no leave when needed among Black workers compare to white workers is driven by differences the returns on demographic, access, and reason coefficients.

## 4. DISCUSSION

The dearth of paid family and medical leave laws leaves women and workers of color without access to leave that is paid and of sufficient duration when facing a qualifying event. We found that women in particular were more likely to take leave of insufficient length or to take no leave at all when need when compared to men. Hispanic workers were more likely to take leave of insufficient length and non-Hispanic Black workers were more likely to take no leave at all when needed relative to white workers. Our paper extends the literature by providing new insight into what drives these differences.

Differences in demographic characteristics, the type of qualifying event, and likely access to paid leave policies or company practices did not explain the increased likelihood of women taking insufficient or no leave when needed. In other words, among similar workers who should have access to the same paid leave policies, women are less likely to take as much leave as they need relative to men. These results suggest that sexism and discrimination play a role in women’s leave-taking, though our data do not allow us to fully untangle their impact. This could manifest through increased real or perceived stigma around leave-taking or fear of retribution for taking leave. Occupational sorting also appears to play a large role for women. Even within service and retail occupations, women may be more likely to work in jobs and firms that do not provide paid leave benefits. However, once occupation and employer were accounted for in our models, women were no longer more likely to take insufficient or no leave relative to men. While our data cannot adjudicate between demand-size and supply-size drivers of this occupational and firm sorting, it is possible that women face barriers at the time of hire to firms that provide the most generous leave policies.

For Hispanic and non-Hispanic Black workers, differences in demographic characteristics and the type of qualifying event explained most of the increase in sufficient leave and not taking any leave when needed. Unlike prior studies, we did not find differential access to paid or sufficient leave among Hispanic workers once demographic characteristics are controlled for. This could be because we focus exclusively within service and retail jobs, where the relevant characteristics are potentially more similar between Hispanic and non-Hispanic white workers. This could also have to do with selection into our sample. Only 8% of our sample identified as Hispanic, compared to 18.7% of the U.S. retail workforce and 17.5% of the total workforce.^48^

Our study uses a novel measure of leave access that takes into account not only whether or not an individual who experienced a qualifying event took leave, but whether the leave was of sufficient duration to meet their needs. We also used a more traditional measure of paid leave, which we report as a supplementary analysis. These results closely mirror the results for leave sufficiency, with paid leave corresponding with sufficient leave and unpaid leave with insufficient leave (the no leave categories are mechanically the same group). Interestingly, while workers reporting sufficient leave were significantly more likely to take paid leave than workers reporting insufficient leave, the correlation between leave sufficiency and leave compensation is lower than one might expect. This suggests that, while pay is an important component of one’s leave-taking experience, other factors also contribute, such as feeling pressure to return to work, fear of losing one’s job, and not knowing about available leave benefits.

### 4.1 Limitations

While the Shift Project data include more details about paid family and medical leave access and use than most other surveys, there remain gaps in what we can explain with our data. We encourage future studies to include additional questions about knowledge of paid leave benefits, perceived stigma around leave-taking, and real or perceived retaliation for taking leave. Additionally, future studies should include even more detailed questions that would enable a deeper understanding of leave-taking behavior, including differentiating respondents with qualifying events who did not take leave because they did not have any paid leave, did not want to use their available leave, or had already used all of their employer-provided leave. Studies drawing on an even larger and more racially diverse sample could examine the interaction between gender and race/ethnicity, which we were insufficiently powered to do. Further, our sample did not include sufficient numbers of respondents who identified as Asian, Pacific Islander, American Indian, or Alaska Native to allow analysis for these subgroups.

## Supplementary Material

Supinfo

## Figures and Tables

**Table I.** Leave Sufficiency and Compensation Type Amongst Survey Respondents

Variable	Percent
Leave Sufficiency	
Sufficient leave	21.2%
Insufficient leave	38.4%
No leave	40.4%
	
Leave Compensation	
Paid leave	19.4%
Unpaid leave	40.2%
No leave	40.4%
	

N	2595

**Table II.** Occupational and Demographic Characteristics of Survey Respondents

Variable	Percent
Gender	
Men	24%
Women	76%
Race	
White, non-Hispanic	85%
Black, non-Hispanic	3%
Hispanic	7%
Other/Multiple	5%
Marital Status	
Married, living with spouse	33%
Living with a partner	19%
Not living with a spouse or partner	48%
Enrolled in School	17%
Parental Status	
Kids 0 to 4	8%
Kids 5 to 9	7%
Kids 10 to 14	10%
Kids 15 to 18	14%
Educational Attainment	
No degree or diploma earned	4%
High school diploma/GED	34%
Some college	38%
Associate’s degree	12%
Bachelor’s degree	10%
Master’s degree/Advanced degree	2%
Work Tenure	
Less than 1 year	18%
1 year	15%
2 years	14%
3 years	10%
4 years	5%
5 years	6%
6 years	3%
7 years	3%
8 years	2%
9 years	2%
10+ years	21%
Union	13%
Leave Reasons	
Health	50%
Caregiving	31%
Multi	19%
Average Age	42
Average Hours Worked/Week	32
Average Hourly Wage	$13.69

N	2595

**Table III.** Estimates of the Association Between Taking Leave and Race/Gender

	(1)	(2)	(3)	(4)	(5)
	Unadjusted	Demographics	Event Types	Access	Occupation/Employer
**Took Leave, Sufficient Length (ref)**					
Men (ref)	0.00	0.00	0.00	0.00	0.00
White, non-Hispanic (ref)	0.00	0.00	0.00	0.00	0.00

**Took Leave, Insufficient Length**					
Men (ref)	0.00	0.00	0.00	0.00	0.00
Women	0.43***	0.35**	0.34**	0.30**	0.21
White, non-Hispanic (ref)	0.00	0.00	0.00	0.00	0.00
Black, non-Hispanic	0.52	0.39	0.35	0.42	0.55
Hispanic	0.44*	0.25	0.20	0.12	0.21
Other/Multiple	0.07	−0.07	−0.12	−0.21	−0.32

**No Leave, Needed Leave**					
Men (ref)	0.00	0.00	0.00	0.00	0.00
Women	0.37**	0.27*	0.29*	0.22	0.04
White, non-Hispanic (ref)	0.00	0.00	0.00	0.00	0.00
Black, non-Hispanic	0.71*	0.56	0.30	0.34	0.54
Hispanic	0.29	−0.00	−0.12	−0.18	−0.09
Other/Multiple	0.15	−0.11	−0.35	−0.39	−0.32

Observations	2595	2595	2595	2595	2595
Month and Year Fixed Effects	✓	✓	✓	✓	✓
Demographic Characteristics		✓	✓	✓	✓
Type of Qualifying Event			✓	✓	✓
Measures of Access/Eligibility				✓	✓
Occupation Fixed Effects					✓
Employer Fixed Effects					✓

*** p<0.001

** p<0.01

* p<0.05

+ p<0.10

**Table IV.** Blinder-Oaxaca Decompositions of Leave Sufficiency by Gender

	Took leave, insufficient length	No leave, wanted leave
	Coef. (s.e.)	Coef. (s.e.)
Differential		
Men	0.569 (0.03)***	0.591 (0.028)***
Women	0.669 (0.015)***	0.678 (0.015)***
Difference	−0.1 (0.034)**	−0.086 (0.031)**
Adjusted	−0.12 (0.087)	−0.094 (0.075)

Endowments		
Demographics	−0.015 (0.008)+	−0.028 (0.009)**
Occupation	−0.034 (0.027)	−0.053 (0.025)*
Access	0.008 (0.015)	−0.002 (0.014)
Reasons	−0.002 (0.002)	−0.008 (0.008)
Total	−0.043 (0.029)	−0.091 (0.029)**

Coefficients		
Demographics	−0.156 (0.333)	−0.066 (0.233)
Occupation	−0.052 (0.081)	−0.012 (0.083)
Access	−0.151 (0.168)	0.193 (0.151)
Reasons	−0.067 (0.032)*	−0.044 (0.016)**
Constant	0.359 (0.39)	−0.048 (0.305)
Total	−0.067 (0.101)	0.024 (0.084)

Interaction		
Demographics	−0.021 (0.021)	0.004 (0.016)
Occupation	0.004 (0.056)	−0.056 (0.049)
Access	0.01 (0.034)	0.029 (0.031)
Reasons	−0.003 (0.004)	−0.003 (0.005)
Total	−0.01 (0.059)	−0.026 (0.047)

*** p<0.001

** p<0.01

* p<0.05

+ p<0.10

**Table V.** Blinder-Oaxaca Decompositions of Leave Sufficiency by Race

	Took leave, insufficient length	No leave, wanted leave
	Coef. (s.e.)	Coef. (s.e.)
Differential		
White, non-Hispanic	0.633 (0.014)***	0.645 (0.014)***
Black, non-Hispanic	0.744 (0.067)***	0.788 (0.057)***
Difference	−0.111 (0.069)	−0.143 (0.059)*
Adjusted	0.097 (0.081)	−0.183 (0.065)**

Endowments		
Demographics	−0.019 (0.069)	−0.03 (0.032)
Occupation	0.021 (0.096)	−0.029 (0.03)
Access	−0.107 (0.091)	0.082 (0.053)
Reasons	0.014 (0.028)	−0.049 (0.028)+
Total	−0.091 (0.077)	−0.026 (0.059)

Coefficients		
Demographics	1.217 (0.121)***	−0.359 (0.088)***
Occupation	−0.087 (0.106)	−0.028 (0.051)
Access	0.415 (0.117)***	−0.361 (0.077)***
Reasons	−0.02 (0.026)	0.061 (0.016)***
Constant	−1.449 (0.127)***	0.525 (0.103)***
Total	0.076 (0.099)	−0.162 (0.071)*

Interaction		
Demographics	−0.004 (0.075)	0.006 (0.036)
Occupation	0.005 (0.103)	0.035 (0.045)
Access	0.128 (0.093)	−0.046 (0.052)
Reasons	−0.017 (0.025)	0.011 (0.012)
Total	0.112 (0.096)	0.005 (0.065)

*** p<0.001

** p<0.01

* p<0.05

+ p<0.10
