# Supplementary material for: Racial/Ethnic and Gender Inequities in the Sufficiency of Paid Leave During the COVID-19 Pandemic: Evidence from the Service Sector Inequities in Paid Leave Sufficiency
Source: Am J Ind Med. Author manuscript; Available in PMC 2023 Nov 28. (PMC10684272; doi:10.1002/ajim.23533)
Supplement: Supinfo [file NIHMS1926249-supplement-Supinfo.docx]

**Appendix**

| **Table A-I**  Firms With Employees Who Reported Experiencing a PFML-Qualifying Event In the 12 Months Preceding Interview. Source: Authors’ analysis of data from the Shift Project Surveys, 2020-2021 | | | |
| --- | --- | --- | --- |
| (1) | (2) | (3) | (4) |
|  |  |  |  |
| 7-Eleven | Dick's Sporting Goods | Jimmy John's | Sams Club |
| Ace Hardware | Disney | Kohls | Shaw's |
| Advance Auto Parts | Dollar General | Kroger/QFC | Sherwin Williams |
| Albertsons | Dollar Tree | LongHorn Steakhouse | ShopRite |
| Aldi | Domino's | Lowe's | Smith's Food and Drug |
| Amazon | Dunkin Donuts | Macy's | Sonic |
| American Eagle | Express | Marriott | Staples |
| Applebees | Fedex | Marshalls | Starbucks |
| Arby's | Food Lion | McDonald's | Stop & Shop |
| AT&T | GameStop | Meijer | Subway |
| Barnes & Noble | Gap | Menards | Taco Bell |
| Bath & Body Works | Giant | Michaels | Target |
| Bed Bath & Beyond | Giant Eagle | O'Reilly Auto Parts | Texas Roadhouse |
| Best Buy | Golden Corral | Office Depot | TGI Friday's |
| Best Western | Hannaford | Olive Garden | Trader Joe's |
| Big Lots | Hardee's | Outback Steakhouse | Ulta Beauty |
| Bob Evans | Harris Teeter | Panera | UPS |
| Buffalo Wild Wings | HEB | Papa John's | Victoria's Secret |
| Burger King | Hilton | Petco | Waffle House |
| Cheesecake Factory | Hobby Lobby | PetSmart | Walgreens |
| Chick-Fil-A | Home Depot | Pizza Hut | Walmart |
| Chili's | Hy-Vee | Price Chopper | Wegmans |
| Chipotle | Hyatt | Publix | Wendy's |
| Costco | IHOP | QuikTrip | Whataburrger |
| Cracker Barrel | Ikea | Red Lobster | Whole Foods |
| CVS | In-N-Out Burgers | Rite Aid | Wyndham |
| Denny's | Jack in the Box | Ross | XPO Logistics |
| DHL | JCPenney | Safeway |  |
|  |  |  |  |

| **Table A-II** Leave Sufficiency by Leave Type. Source: Authors’ analysis of data from the Shift Project Surveys, 2020-2021 | | | | |
| --- | --- | --- | --- | --- |
|  |  |  |  |  |
|  | Leave Compensation | | |  |
|  | Paid leave | Unpaid leave | No leave | Total |
| Leave Sufficiency |  |  |  |  |
| Sufficient leave | 218 (39.6%) | 333 (60.4%) | 0 (0%) | 551 (100%) |
| Insufficient leave | 285 (28.6%) | 711 (71.4%) | 0 (0%) | 996 (100%) |
| No leave | 0 (0%) | 0 (0%) | 1048 (100%) | 1048 (100%) |

| **Table A-III** Leave Sufficiency by Leave Type. Source: Authors’ analysis of data from the Shift Project Surveys, 2020-2021 | | | | | |
| --- | --- | --- | --- | --- | --- |
|  | (1) | (2) | (3) | (4) | (5) |
|  | Unadjusted | Demographics | Event Types | Access | Occupation |
| **Paid Leave** |  |  |  |  |  |
| Men (ref) | 0.00 | 0.00 | 0.00 | 0.00 | 0.00 |
| White, non-Hispanic (ref) | 0.00 | 0.00 | 0.00 | 0.00 | 0.00 |
| **Unpaid Leave** |  |  |  |  |  |
| Men (ref) | 0.00 | 0.00 | 0.00 | 0.00 | 0.00 |
| Women | 0.50*** | 0.43*** | 0.44*** | 0.23+ | 0.24 |
| White, non-Hispanic (ref) | 0.00 | 0.00 | 0.00 | 0.00 | 0.00 |
| Black, non-Hispanic | 0.51 | 0.42 | 0.40 | 0.46 | 0.83* |
| Hispanic | 0.13 | 0.07 | 0.04 | 0.21 | 0.28 |
| Other/Multiple | -0.10 | -0.17 | -0.20 | -0.16 | -0.17 |
| **No Leave** |  |  |  |  |  |
| Men (ref) | 0.00 | 0.00 | 0.00 | 0.00 | 0.00 |
| Women | 0.44*** | 0.33** | 0.36** | 0.18 | 0.05 |
| White, non-Hispanic (ref) | 0.00 | 0.00 | 0.00 | 0.00 | 0.00 |
| Black, non-Hispanic | 0.72* | 0.59 | 0.36 | 0.38 | 0.74+ |
| Hispanic | 0.08 | -0.13 | -0.24 | -0.12 | -0.03 |
| Other/Multiple | 0.03 | -0.18 | -0.41 | -0.36 | -0.22 |
| Observations | 2595 | 2595 | 2595 | 2595 | 2595 |
| Month and Year Fixed Effects | ✓ | ✓ | ✓ | ✓ | ✓ |
| Demographic Characteristics |  | ✓ | ✓ | ✓ | ✓ |
| Type of Qualifying Event |  |  | ✓ | ✓ | ✓ |
| Measures of Access/Eligibility |  |  |  | ✓ | ✓ |
| Occupation FE |  |  |  |  | ✓ |
| Employer Fixed Effects |  |  |  |  | ✓ |
| ***p<0.001, **p<0.01, *p<0.05, +p<0.10 | | | | | |
